# Digital Fabrication of an Occlusal Reference Index to Facilitate Crown Insertion and Minimize Unnecessary Occlusal Adjustments in Case of Unstable Occlusion: A Clinical Report

**DOI:** 10.1155/crid/5769281

**Published:** 2026-08-04

**Authors:** Abdulaziz M. Alqarni, Abdulaziz Fahmy Flimban, Ahmad Y. Imam, Thamer Y. Marghalani, Alireza Moshaverinia

**Affiliations:** ^1^ Department of Prosthodontics, King Fahad General Hospital, Ministry of Health, Jeddah, Saudi Arabia, moh.gov.sa; ^2^ Department of Oral and Maxillofacial Prosthodontics, Faculty of Dentistry, King Abdulaziz University, Jeddah, Saudi Arabia, kau.edu.sa; ^3^ Private practice, Los Angeles, California, USA

**Keywords:** crown insertion, malocclusion, occlusal accuracy, occlusal adjustment, prosthodontic technique

## Abstract

Accurate occlusal assessment during single crown insertion can be compromised by proprioceptive misperception, leading to unnecessary occlusal adjustment. The report introduces a digitally fabricated occlusal index that serves as a stable occlusal reference during crown insertion. This method may help to minimize the risk of overadjustment and enhances chairside efficiency.

## 1. Introduction

The clinical success and longevity of dental restorations are fundamentally dependent on occlusal design [1]. A harmonious occlusion ensures functional integration within the stomatognathic system, promoting even force distribution and minimizing destructive stresses. Conversely, occlusal discrepancies create static and dynamic interferences, which are primary etiological factors in restoration failure [1, 2]. Improper occlusion on crowns or fixed dental prosthesis (FDP), particularly high contacts in maximum intercuspal position (MIP), can cause biological and functional complications [3]. Teeth may shift under excessive force, and patients may develop temporomandibular joint (TMJ) clicking or jaw discomfort. If uncorrected, high restorations may lead to mobility, hypersensitivity, pain, muscle fatigue, and, in more advanced situations, loss of supporting bone [3]. Therefore, achieving precise occlusion at cementation is a common clinical challenge, often necessitating postcementation adjustments to ensure functional harmony [4–6].

Accurate occlusal evaluation is essential during crown insertion. Ultrathin indicators, such as Shimstock and articulating film of ≤ 21 *μ*m, are recommended to ensure precise contact assessment [7]. In contrast, using thicker articulating papers may alter the patient′s natural closure by stimulating proprioception and increasing bite force, potentially leading to mandibular deflection [8–10]. This may misleadingly indicate a high occlusion, inaccurate occlusal markings, and potentially prompt the clinician to make unnecessary adjustments [9, 10]. This problem is exacerbated when a clinician lacks a clear bite reference from the opposing teeth, or when a preexisting malocclusion makes it difficult to find a stable, reliable guide for the correct occlusal relationship. With the evolution of digital dental workflows, this case report presents a practical technique to fabricate a simple occlusal reference index that may help to reduce proprioceptive interference, improving insertion procedure and efficiency by limiting unnecessary postcementation adjustments.

## 2. Case Report

A 56‐year‐old male presented to the prosthodontic clinic for restoring an endodontically treated tooth with full coverage restoration. Medical history was unremarkable, with no significant findings or regular medication use. Clinical examination of the remaining dentition revealed an anterior open bite, unilateral cross‐bite on the right side, crowding in both the maxillary and mandibular arches, generalized occlusal tooth wear, multiple restorations, and generalized Stage I, Grade A periodontitis, characterized by a mean probing depth of less than 4 mm. During occlusion evaluation, an unstable bite was noted. The tooth was then prepared, and a digital workflow was processed.

### 2.1. Patient Consent

The patient provided written informed consent for both photographic documentation and the publication of the case.

### 2.2. Technique

**Figure 1:**
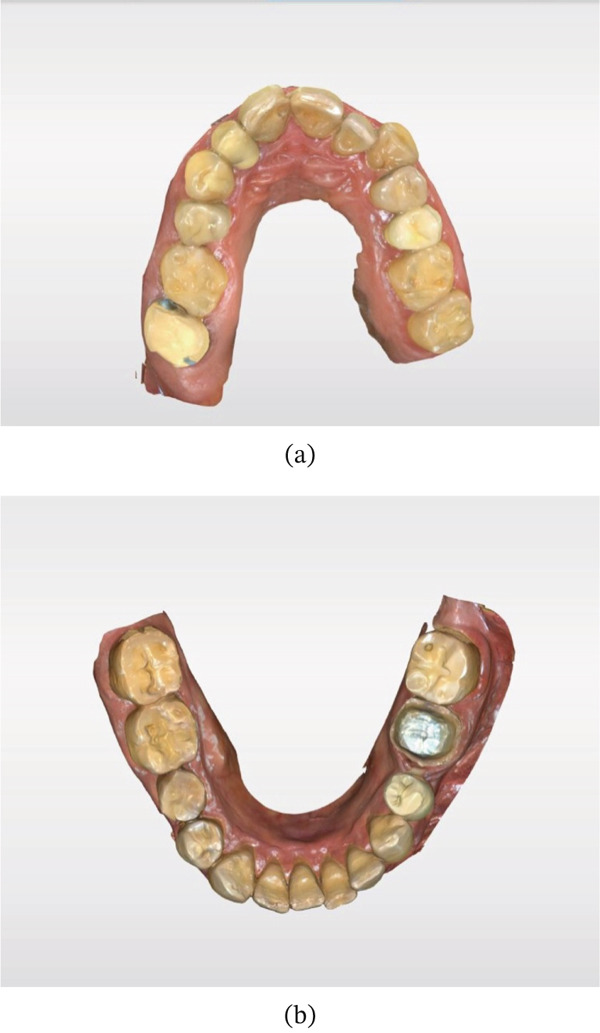
Scan arches obtained with an intraoral scanner (Primescan; Dentsply Sirona, Bensheim, Germany). (a) Maxillary arch. (b) Mandibular arch.

**Figure 2:**
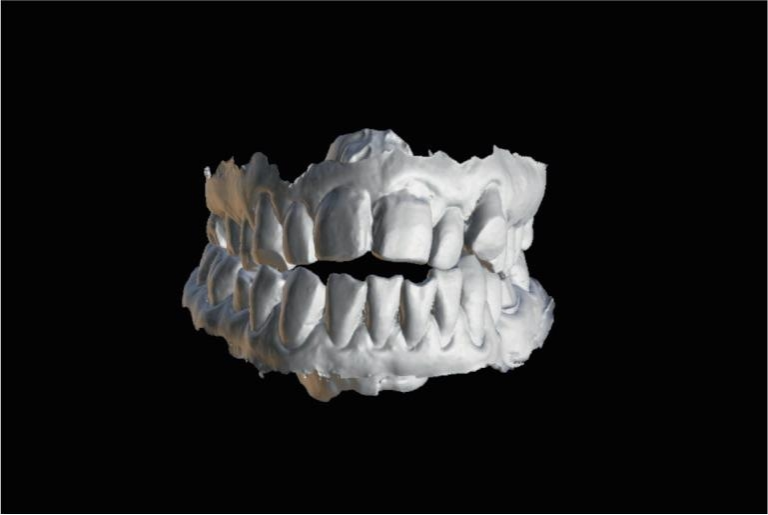
Aligned maxillary and mandibular scans using a dental software program (Blue Sky Plan 4.0; Blue Sky Bio, Libertyville, IL) to create a virtual articulated model.

**Figure 3:**
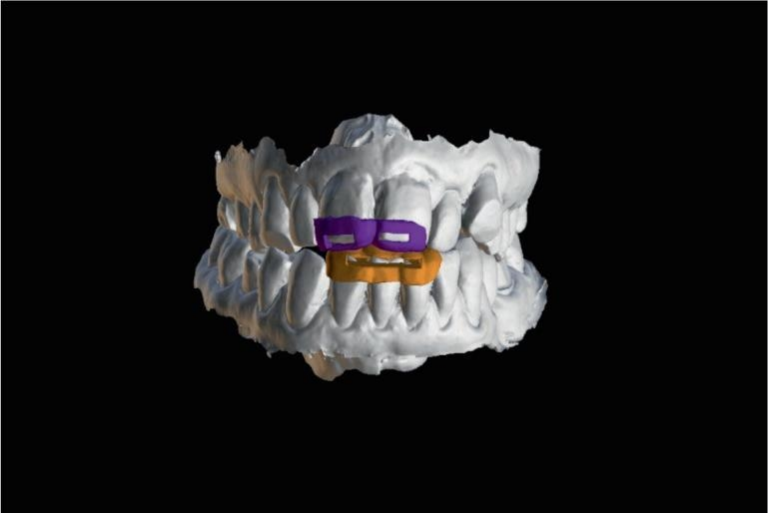
Digitally designed occlusal index covering incisal and partial facial/lingual contours with verification windows and thickness maintained at 1.0–1.5 mm.

The maxillary and mandibular arches were scanned using an intraoral scanner (Primescan; Dentsply Sirona, Bensheim, Germany), and right and left virtual bite records were obtained in MIP, including the prepared tooth (Figure 1). The scan files were imported into a dental software program (Blue Sky Plan 4.0; Blue Sky Bio, Libertyville, IL) and aligned to generate a single virtual articulated model (Figure 2). An occlusal reference index was then designed using the guide panel to cover the incisal surfaces and partial facial and lingual contours of the maxillary and mandibular anterior teeth. Verification windows were incorporated to facilitate confirmation of complete seating, and a minimum index thickness of 1.0–1.5 mm was maintained to provide adequate rigidity while preventing occlusal interference (Figure 3).

**Figure 4:**
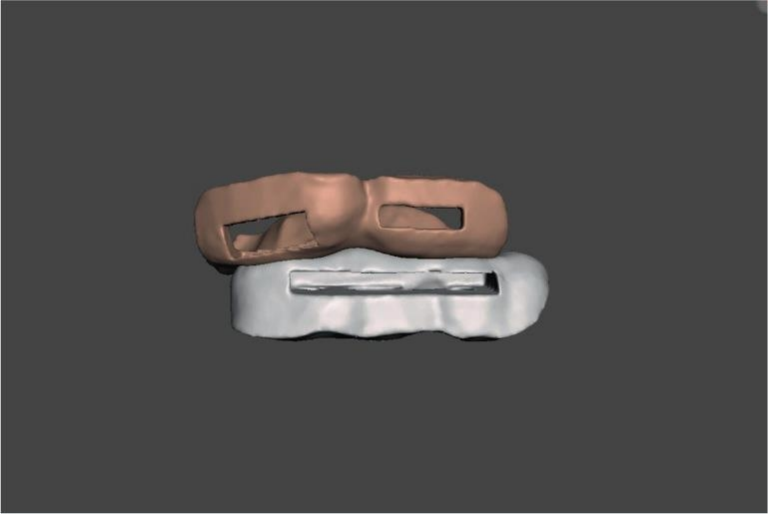
Occlusal index file imported and refined in 3D design software program (Meshmixer 3.5; Autodesk Inc, San Rafael, CA) using Sculpt tool.

The finalized index design was exported as a standard tessellation language (STL) file and imported into a three‐dimensional (3D) design software program (Meshmixer 3.5; Autodesk Inc., San Rafael, CA). The files were combined, and the Sculpt tool was used to contour and refine the index before exporting the final design for additive manufacturing (Figure 4). The index was fabricated using a digital light processing 3D printer (Asiga Max; Asiga, Alexandria, Australia) with a biocompatible resin (NextDent SG; NextDent, Soesterberg, Netherlands) at a 0‐degree build orientation and a 100‐*μ*m‐layer thickness. After printing, the supports were removed, the index was cleaned in isopropyl alcohol for 5 min, rinsed with water, and postcured in a curing unit (ProCure; SprintRay, Los Angeles, CA).

**Figure 5:**
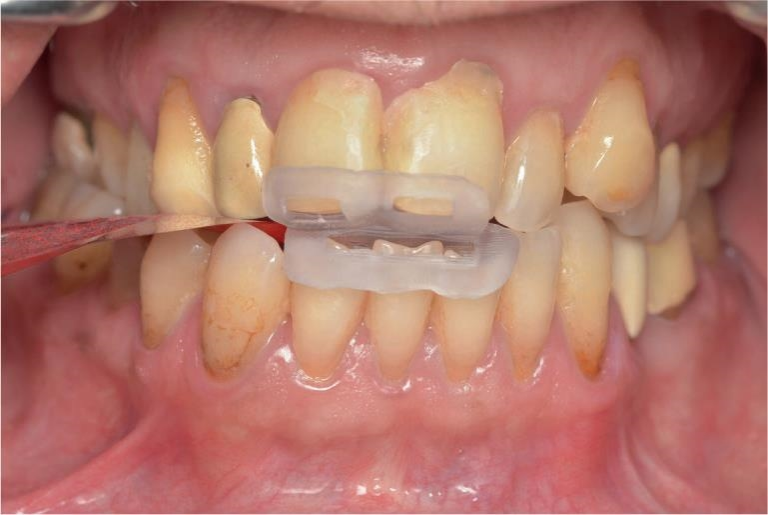
Try‐in of fabricated occlusal index showing passive fit and verified occlusal clearance with 12‐*μ*m shimstock (Bausch GmbH & Co. KG, Köln, Germany) at maximum intercuspal position.

The printed index was tried intraorally to verify adaptation and passive fit. Occlusal clearance was assessed on the right posterior teeth using 12‐*μ*m shimstock (Bausch GmbH & Co. KG, Köln, Germany) before placing the index. The patient was then instructed to close into MIP with the index seated passively, and the same occlusal contact was confirmed to ensure that the index did not introduce occlusal interference (Figure 5). The definitive monolithic zirconia crown (3D Pro Zirconia; Aidite Technology Co., Ltd., Qinhuangdao, China), fabricated using a five‐axis milling machine (Ceramill Motion 3; Amann Girrbach, Koblach, Austria), was placed on the prepared tooth and evaluated for proximal contacts, marginal adaptation, and overall stability to confirm complete seating.

The occlusal reference index was repositioned, and the patient was instructed to close gently until intimate contact with the index was achieved. Occlusal contacts in MIP were evaluated using 40‐*μ*m articulating paper (Bausch GmbH & Co. KG, Köln, Germany), and any required occlusal adjustments were verified. The index was then removed, and eccentric movements were evaluated to confirm smooth lateral and protrusive guidance. The zirconia crown was subsequently finished and polished using zirconia rubber polishers (DIACERA Kit; EVE Ernst Vetter GmbH, Pforzheim, Germany).

**Figure 6:**
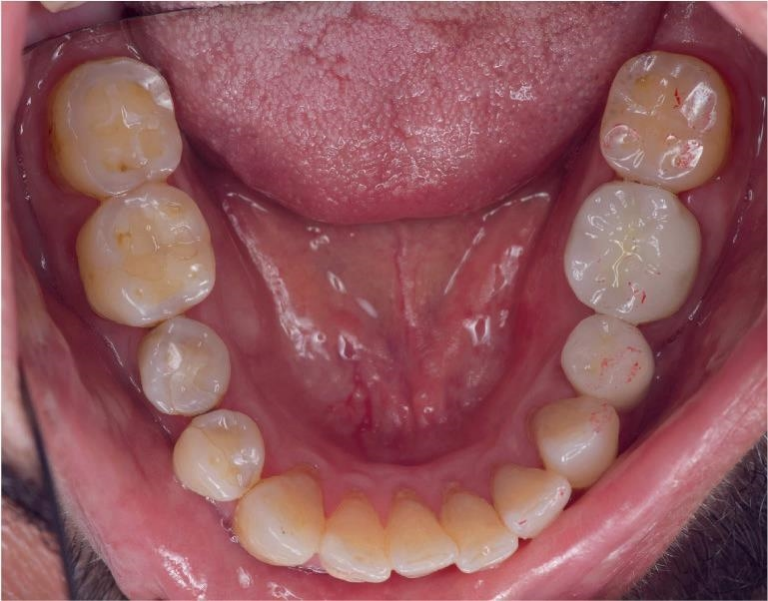
Occlusal view of cemented zirconia crown.

Finally, occlusion was verified in centric and eccentric movements. The tooth was isolated, cleaned, and dried, and the intaglio surface of the restoration was treated with airborne‐particle abrasion using 50‐*μ*m alumina particles. A methacryloyloxydecyl dihydrogen phosphate (MDP)–containing primer (Panavia V5; Kuraray Noritake Dental Inc., Tokyo, Japan) was applied, and the crown was cemented with a dual‐cure resin cement (Panavia V5; Kuraray Noritake Dental Inc., Tokyo, Japan). Excess cement was carefully removed, and complete seating of the restoration was confirmed (Figure 6).

The patient was recalled for follow‐up assessments at 72 h and at 1 month postdelivery. At both intervals, the patient remained asymptomatic, with no reported complaints or adverse findings. Clinical examination revealed satisfactory restoration integrity, stable occlusion, and healthy soft tissue response.

## 3. Disscussion

The application of an occlusal index at the time of crown insertion serves to minimize unnecessary occlusal adjustment of accurately contoured restorations, particularly in patients with malocclusion. Patient‐related proprioceptive interference during the try‐in phase may cause a guarded or altered mandibular closure, often leading to false high‐spot identification and unwarranted occlusal reduction [10]. This challenge is further amplified in patients with existing malocclusion, such as anterior open bite, crossbite, or excessive horizontal overjet, where establishing a repeatable and stable intercuspal position may be difficult.

The presented technique introduces a digitally fabricated occlusal reference index to provide a reproducible and objective occlusal guide during crown delivery. The index is designed when there is sufficient occlusal space to allow accurate fabrication and prevent interference with functional surfaces, which is especially important in cases of open bite, crossbite, or other malocclusions. Additionally, the technique is particularly valuable in clinical situations involving dyskinesia, unstable occlusion, patients with poor neuromuscular control, or patients with special needs, where maintaining consistent mandibular positioning can be challenging. The available space facilitates digital design by accommodating appropriate material thickness, allows clear clinical visualization, and supports passive seating of the restoration while guiding the patient into the intended maximum intercuspation position for which the crown was fabricated. By strategically positioning the index in an area with adequate clearance, the risk of generating inaccurate occlusal contacts or introducing mandibular positioning errors is minimized.

Shimstock is ideal for detecting whether contact exists, whereas articulating paper identifies contact location, but both rely on patient‐driven closure and may be influenced by proprioceptive bias, especially during crown try‐in [9–12]. The digitally fabricated occlusal reference index may provide an independent, stable closure guide, improving accuracy by distinguishing true occlusal discrepancies from proprioceptive interference.

However, when digital equipment is not available, conventional methods remain practical alternatives. Polyvinyl siloxane (PVS) bite registration materials and autopolymerizing acrylic resins may be used to fabricate a comparable occlusal index chairside or indirectly through a dental laboratory. These materials provide the benefit of immediate fabrication without scanning or printing requirements, making them suitable for conventional workflows.

Limitations of the digital method include the need for intraoral scanning, 3D printing, and software proficiency, which may increase treatment costs and may not be available in all clinical settings. Minor distortions during printing or post‐processing can affect index seating, and patient‐specific anatomical variations or malocclusion may complicate placement of such occlusal guidance. In addition, although the proposed technique may facilitate crown seating and reduce proprioceptive interference, further clinical studies are needed to confirm these potential benefits and evaluate its clinical effectiveness, cost‐effectiveness, and long‐term outcomes.

## 4. Conclusion

This clinical report employs a digitally fabricated occlusal index to help verify patient occlusion, identify true high contacts, and may help reduce unnecessary adjustments and chairside time during crown try‐in, offering improved accuracy, efficiency, and a more predictable seating process while minimizing the risk of repeated occlusal corrections and enhancing overall clinical workflow.

## Funding

No funding was received for this manuscript.

## Conflicts of Interest

The authors declare no conflicts of interest.

## Data Availability

The data that support the findings of this study are available from the corresponding author upon reasonable request.
